# Age-Related Differences in Antihypertensive Medication Adherence in Hispanics: A Cross-Sectional Community-Based Survey in New York City, 2011–2012

**DOI:** 10.5888/pcd14.160512

**Published:** 2017-07-13

**Authors:** Priti Bandi, Emily Goldmann, Nina S. Parikh, Parisa Farsi, Bernadette Boden-Albala

**Affiliations:** 1 College of Global Public Health, New York University, New York, New York; 2 Department of Neurology, School of Medicine, Langone Medical Center, New York University, New York, New York; 3 Department of Epidemiology and Health Promotion, College of Dentistry, New York University, New York, New York

## Abstract

**Introduction:**

US Hispanics, particularly younger adults in this population, have a higher prevalence of uncontrolled hypertension than do people of other racial/ethnic groups. Little is known about the prevalence and predictors of antihypertensive medication adherence, a major determinant of hypertension control and cardiovascular disease, and differences between age groups in this fast-growing population.

**Methods:**

The cross-sectional study included 1,043 community-dwelling Hispanic adults with hypertension living in 3 northern Manhattan neighborhoods from 2011 through 2012. Age-stratified analyses assessed the prevalence and predictors of high medication adherence (score of 8 on the Morisky Medication Adherence Scale [MMAS-8]) among younger (<60 y) and older (≥60 y) Hispanic adults.

**Results:**

Prevalence of high adherence was significantly lower in younger versus older adults (24.5% vs 34.0%, *P* = .001). In younger adults, heavy alcohol consumption, a longer duration of hypertension, and recent poor physical health were negatively associated with high adherence, but poor self-rated general health was positively associated with high adherence. In older adults, advancing age, higher education level, high knowledge of hypertension control, and private insurance or Medicare versus Medicaid were positively associated with high adherence, whereas recent poor physical health and health-related activity limitations were negatively associated with high adherence.

**Conclusion:**

Equitable achievement of national hypertension control goals will require attention to suboptimal antihypertensive medication adherence found in this study and other samples of US Hispanics, particularly in younger adults. Age differences in predictors of high adherence highlight the need to tailor efforts to the life stage of people with hypertension.

## Introduction

Hypertension affects approximately one-third of the US adult population and is a leading risk factor for cardiovascular disease (CVD), including heart disease, stroke, and chronic kidney disease (1). Proper treatment and control of hypertension prevents first and recurrent CVD, but less than half of people with hypertension in the United States have achieved blood pressure control (2). Significant hypertension-related disparities have been documented in Hispanics, the largest and fastest-growing racial/ethnic group in the United States, leaving this population at risk for disproportionately negative CVD outcomes. For example, Sorlie et al reported that only 38% of Hispanics had controlled hypertension, an important determinant of CVD risk, compared with 56% of non-Hispanic whites (3). Furthermore, only 74% of Hispanics were aware of their hypertension status compared with 81% of non-Hispanic whites, and 63% of Hispanics were treated for this condition compared with 77% of non-Hispanic whites (3).

Antihypertensive medication adherence, defined as the extent of correspondence between a person’s medication-related behavior and health care provider recommendations, is an important determinant of hypertension control and improved CVD outcomes (4,5). On the basis of literature that identifies predictors of adherence in the general population, the World Health Organization (WHO) Multidimensional Adherence Model classifies predictors of adherence among 5 multilevel, domains, including health care system–, patient-, socioeconomic-, therapy-, and condition-related (4–6). Although Hispanics have lower levels of adherence compared with non-Hispanic whites, little is known about predictors of adherence in this group (2,7,8). Addressing ethnic disparities in hypertension control and CVD outcomes requires a better understanding of predictors of adherence in these populations.

Although adherence to long-term therapies is known to improve with age, predictors of adherence are not necessarily similar between older and younger groups (5,7,9). In general, young adults with hypertension have suboptimal hypertension outcomes (1), which is particularly relevant for Hispanics, who have significantly higher rates of hypertension among younger adults than do non-Hispanic whites of the same age (1). Identifying age-related differences in predictors of adherence among Hispanics is important to effectively target factors responsible for documented age-related and ethnic disparities in hypertension outcomes in the United States.

To address this gap in the literature, we evaluated the prevalence and predictors of antihypertensive medication adherence in younger and older adults separately, using a large sample of community-dwelling Hispanic adults with hypertension in New York City. Because adherence is a complex behavior simultaneously influenced by multiple individual and environmental factors, we grounded our assessment in the WHO Multidimensional Adherence Model framework (4,6). We discuss the implications of our findings for addressing ethnicity- and age-based disparities in adherence and hypertension control.

## Methods

### Data and analytic sample

Data came from the Washington Heights/Inwood Informatics Infrastructure for Community-Centered Comparative Effectiveness Research (WICER) project (10,11). Briefly, residents of 3 low-income, primarily immigrant northern Manhattan neighborhoods, recruited through a probability sample of households and snowball sampling, were asked to participate in an in-person cross-sectional survey administered by trained, bilingual (Spanish and English) community health workers from March 2011 through November 2012. Household members aged 18 years or older were eligible to participate and were offered local grocery store food vouchers for their time. Informed consent was obtained before survey administration, and the study was approved by the Columbia University Medical Center institutional review board.

This study’s analytic sample comprised respondents who self-identified as being Hispanic, Latino, or of Spanish origin, reported a diagnosis of high blood pressure or hypertension by a health care provider, and were currently taking medication for this condition (N = 1,043). The sample was categorized as younger (<60 y, 41.1% [n = 429]) and older adults (≥60 y, 58.9%, [n = 614]) to reflect the natural distribution of age in this sample (mean age, 61.9 y) and based on age-specific (<60 y, ≥60 y) guidelines for the management of hypertension in adults (12).

### Measures

Antihypertensive medication adherence was assessed using the self-reported 8-item Morisky Medication Adherence Scale (MMAS-8), which has been validated against blood pressure levels and shown to be valid in a sample of primarily low-income, minority patients with hypertension (13). The scale consisted of 8 dichotomous (yes/no, with no coded as 1, indicating higher adherence) or Likert items (normalized to range between 0 and 1, with higher values indicating higher adherence). Examples of items were, “Do you ever forget to take your high blood pressure medication?” and **“**When you feel like your blood pressure is under control, do you ever stop taking your medicine?” (13). Recoded items were summed and ranged from 0 to 8. Internal consistency, measured using Cronbach α, was good (α = 0.73). The summed score was dichotomized into high adherence (score of 8) and low or medium adherence (scores of 0 to <8) based on recommended cut points (13).

### Potential predictors

Demographic characteristics were age in years, sex, and marital status (divorced/separated/unmarried vs married or cohabiting). Because 97% of the sample were first-generation immigrants born outside of the United States, most in the Dominican Republic, acculturation measures included length of stay in the United States (living in the United States for <10 years vs living in the United States ≥10 years or US-born) and language of interview (English vs Spanish). Available survey data on potential correlates were selected and classified according to WHO Multidimensional Adherence Model domains (socioeconomic-, health care system–, patient-, and condition-related) (4). Therapy-related factors were not available in the survey and were therefore not included in the assessment.

Socioeconomic or health care system–related factors included education level (≤8th grade, some high school [no diploma], high school diploma/GED, >high school diploma), health insurance status (any Medicaid including other state- or city-sponsored free or low-cost plans, private insurance or Medicare only, or uninsured), current employment status (employed vs unemployed), household composition (lives with >2 people vs lives with ≤2 people or alone), and computer literacy (yes/no), which was measured as use of any social networking sites such as Facebook, MySpace, or Twitter (10).

Patient-related factors included hypertension control knowledge and behavioral risk factors. Hypertension control knowledge was measured using a series of 10 yes/no questions on ways people can treat or control high blood pressure (eg, taking medication, controlling/losing weight). Items were summed into a score that ranged from 0 to 10 and categorized into high (above median) versus low (at or below median). Behavioral risk factors included body mass index (BMI, kg/m^2^) categories estimated from interviewer-measured height and weight (normal weight, <25.0; overweight, 25.0–29.9; or obese, >30.0), smoking status (current smoker vs never or former smoker), number of alcoholic drinks on a typical occasion (0, 1 drink/d, ≥2 drinks/d), and the Centers for Disease Control and Prevention’s (CDC’s) Behavioral Risk Factor Surveillance System 30-day physical activity measure (active defined as moderate or vigorous aerobic physical activity for at least 10 minutes in past month, dichotomized into active or inactive) (14).

Condition-related factors included duration of hypertension (<5 y, 5 to <10 y, ≥10 y) calculated by subtracting respondents’ age at survey and age of hypertension diagnosis, and number of comorbid conditions (none, 1, or ≥2, based on health care provider’s diagnosis of heart disease, stroke, weak or failing kidneys, cancer, or diabetes). The 4-item CDC Health-Related Quality of Life (HRQOL) scale assessed different HRQOL domains, including self-rated general health status (fair/poor, good, very good/excellent), recent (past 30-day) poor physical health (number of physically unhealthy days due to physical illness or injury), poor mental health (number of mentally unhealthy days due to stress, depression, and problems with emotions), and poor health–related activity limitation (number of days usual activities [ie, self-care, work, or recreation] were limited because of poor physical or mental health) (14). Reported days per month on the latter 3 items were dichotomized as yes (above median) versus no (at or below median) due to low observed frequency of the recommended categorization of 14 or more days versus fewer than 14 days (15). 

### Statistical analysis

All analyses were stratified by age group to evaluate the prevalence and predictors of high antihypertensive medication adherence separately for younger adults (<60 y) and older adults (≥60 y). Sample characteristics were compared between younger and older adults using frequencies and means. Age-group specific bivariable analyses (χ^2^ test for categorical variables and standard 2-tailed *t* tests for continuous variables) assessed the relationship between each of the potential predictors and high prevalence of medication adherence. To identify independent predictors of high adherence, a priori–decided variables (demographics and education level) and those associated with high adherence at the *P* = .10 level in bivariable analyses were included in multivariable Poisson regression models with log link function and robust variance, as has been recommended for binary outcomes with high prevalence (16). Adjusted prevalence ratios (APRs) and 95% confidence intervals (CIs) are presented. Multicollinearity was ruled out, because the variance inflation factors of predictors in linear regression models were all less than 10. All analyses were conducted using Stata version 14 (StataCorp LP).

## Results

Compared with older adults, younger adults were significantly more likely to have lived in the United States for less than 10 years, use English as the interview language, be married or cohabiting, and have a higher education level (all *P* < .05); no significant differences were evident for sex (Table 1). Supplementary Table 1 in the Appendix presents a comparison between age groups for all study variables. In the entire sample, the prevalence of high adherence was 30.1%. Younger adults had significantly lower prevalence of high adherence compared with older adults (24.5% vs 34.0%, *P* = .001) (Table 1).

**Table 1:** Selected Sociodemographic Characteristics and High Antihypertensive Medication Adherence Prevalence Among Hispanic Adults With Hypertension by Age Group, WICER Survey, New York City, 2011–2012

Characteristic	Younger Adults (<60 y)	Older Adults (≥60 y)	*P* Value^a^
**Total, % (n)**	41.1 (429)	58.9 (614)	—
**High medication adherence, % (n)^b^**	24.5 (105)	34.0 (209)	.001
**Mean age, y (SD)**	51.7 (6.9)	69.1 (7.0)	<.001
**Sex, % (n)**			
Male	26.3 (113)	23.6 (145)	.32
Female	73.7 (316)	76.4 (469)	
**Immigration status, % (n)**			
Living in United States for <10 y	13.1 (54)	7.9 (46)	.007
Living in United States ≥10 y or US-born	86.9 (357)	92.1 (537)	
**Language of interview, % (n)**			
English	31.2 (134)	23.8 (146)	.007
Spanish	68.8 (295)	76.2 (468)	
**Marital status, % (n)**			
Divorced/separated/unmarried	59.1 (251)	66.9 (409)	.009
Married or cohabiting	40.9 (174)	33.1 (202)	
**Education level, % (n)**			
≤8th grade	34.7 (146)	66.2 (397)	<.001
Some high school (no diploma)	21.9 (92)	13.7 (82)	
High school diploma/GED	20.0 (84)	10.2 (61)	
>High school diploma	23.5 (99)	10.0 (60)	

Abbreviations: —, not applicable; GED, general educational development test; SD, standard deviation; WICER, Washington Heights/Inwood Informatics Infrastructure for Community-Centered Comparative Effectiveness Research.

a *P* value determined by χ^2^ test for categorical variables and standard 2-tailed *t* test for continuous variables.

b High adherence (score of 8) versus low adherence (scores of 0 to <8) on 8-item Morisky Medication Adherence Scale (MMAS-8).

Table 2 presents bivariable and multivariable associations between study variables and high adherence prevalence.

**Table 2:** Age-Specific Bivariable and Multivariable Predictors of High Antihypertensive Medication Adherence^a^ Prevalence Among Hispanic Adults With Hypertension, WICER Survey, New York City, 2011–2012

Characteristic	Younger Adults (<60 y)				Older Adults (≥60 y)			
	Value	*P* Value^b^	APR^c^ (95% CI)	*P* Value	Value	*P* Value^b^	APR^c^ (95% CI)	*P* Value
**Demographics**								
**Mean age, y (SD)**	52.0 (6.8) vs 51.6 (6.9)	.65	1.02 (0.99–1.06)	.18	69.7 (7.2) vs 68.7 (6.9)	.05	1.02 (1.00–1.04)	.02
**Sex, %**								
Male	22.1	.50	1 [Reference]	.70	36.6	.47	1 [Reference]	.56
Female	25.3		0.93 (0.64–1.36)		33.3		0.92 (0.71–1.21)	
**Immigration status, %**								
Living in United States <10 y	25.9	.80	1 [Reference]	.58	39.1	.46	1 [Reference]	.16
Living in United States ≥10 y or US-born	24.4		0.87 (0.54–1.41)		33.7		0.75 (0.50–1.12)	
**Language of interview, %**								
English	20.9	.25	1 [Reference]	.11	28.1	.08	1 [Reference]	.53
Spanish	26.1		1.42 (0.92–2.19)		35.9		1.13 (0.78–1.64)	
**Marital status, %**								
Divorced/separated/unmarried	25.5	.55	1 [Reference]	.41	33.5	.78	1 [Reference]	.46
Married or cohabiting	23.0		0.85 (0.57–1.26)		34.7		1.10 (0.85–1.42)	
**Socioeconomic/Health Care–Related Factors**								
**Education level, %**								
≤8th grade	24.7	.40	1 [Reference]	—	29.2	.01	1 [Reference]	—
Some high school (no diploma)	18.5		0.67 (0.38–1.18)	.17	40.2		1.33 (0.92–1.92)	.13
High school diploma/GED	28.6		1.01 (0.61–1.68)	.96	41.0		1.18 (0.79–1.77)	.41
>High school diploma	27.3		1.06 (0.66–1.69)	.82	46.7		1.51 (1.06–2.14)	.02
**Insurance status, %**								
Any Medicaid^d^	23.3	.14	—	—	31.4	<.001	1 [Reference]	—
Private or Medicare only	22.0		—	—	57.1		1.67 (1.18–2.38)	.004
Uninsured	37.8		—	—	41.2		1.78 (0.86–3.70)	.12
**Current employment status, %**								
Employed	22.9	.56	—	—	28.1	.04	1 [Reference]	.62
Unemployed	25.5		—	—	36.3		0.93 (0.69–1.25)	
**Household composition, %**								
Lives with ≤2 people or alone	27.3	.20	—	—	33.3	.58	—	—
Lives with >2 people	22.0		—	—	35.4		—	—
**Computer literacy,^e^ %**								
No	22.8	.01	1 [Reference]	.07	32.2	.003	1 [Reference]	.27
Yes	41.7		1.64 (0.97–2.78)		57.6		1.27 (0.83–1.95)	
**Patient-Related Factors**								
**Blood pressure control knowledge score,^f^ %**								
Low	23.5	.58	—	—	24.4	<.001	1 [Reference]	<.001
High	25.8		—	—	46.3		1.70 (1.30–2.22)	
**Weight status (body mass index, kg/m^2^), %**								
Normal weight (<25.0)	18.5	.52	—	—	38.2	.37	—	—
Overweight (25.0–29.9)	24.5		—	—	35.3		—	—
Obese (>30.0)	26.1		—	—	31.2		—	—
**Alcohol consumption on typical occasion, %**								
Nondrinker	27.5	.08	1 [Reference]	—	33.1	.84	—	—
1 drink/d	23.1		0.78 (0.46–1.30)	.34	34.9		—	—
≥2 drinks/d	16.4		0.58 (0.35–0.95)	.03	36.5		—	—
**Smoking status, %**								
Never/former smoker	22.7	.003	1 [Reference]	.75	34.3	.72	—	—
Current smoker	46.7		1.11 (0.60–2.05)		38.1		—	—
**Physical activity**								
Inactive	23.5	.21	—	—	32.6	.007	1 [Reference]	.06
Active	31.5		—	—	54.1		1.40 (0.99–1.98)	
**Condition-Related Factors**								
**Duration with hypertension, y, %**								
<5	34.4	.03	1 [Reference]	—	30.3	.60	—	—
5 to <10	26.7		0.63 (0.39–1.02)	.06	37.8		—	—
≥10	19.9		0.54 (0.34–0.84)	.006	32.7		—	—
**Comorbid conditions, %**								
None	27.4	.10	1 [Reference]	—	35.4	.64	—	—
1	17.7		0.69 (0.43–1.11)	.13	31.8		—	—
≥2	28.6		0.87 (0.47–1.63)	.67	31.4		—	—
**Poor physical health, 30-day, %^g^**								
No	31.9	<.001	1 [Reference]	.01	42.7	<.001	1 [Reference]	<.001
Yes	14.1		0.53 (0.33–0.87)		20.3		0.51 (0.35–0.74)	
**Poor mental health, 30-day, %^g^**								
No	26.6	.06	1 [Reference]	.18	38.5	<.001	1 [Reference]	.63
Yes	16.0		0.63 (0.32–1.24)		17.0		0.86 (0.46–1.59)	
**Poor health–related activity limitation, 30-day, %^g^**								
No	25.8	.34	—	—	38.5	<.001	1 [Reference]	<.001
Yes	20.0		—	—	12.8		0.35 (0.17–0.70)	
**Self-rated health, %^g^**								
Very good/excellent	17.4	<.001	1 [Reference]	—	31.3	.17	—	—
Good	31.3		1.45 (0.92–2.28)	.11	37.4		—	—
Fair/poor	38.6		2.37 (1.54–3.63)	<.001	35.8		—	—

Abbreviations: —, not applicable; APR, adjusted prevalence ratio; CI, confidence interval; GED, general educational development test; SD, standard deviation; WICER, Washington Heights/Inwood Informatics Infrastructure for Community-Centered Comparative Effectiveness Research.

a High adherence (score of 8) and low or medium (scores of 0 to <8) on 8-item Morisky Medication Adherence Scale (MMAS-8). Only demographics, education level, and variables associated with high adherence at the *P* = .10 level in bivariate analyses were included in multivariate analyses.

b *P* value determined by using χ^2^ test for categorical variables and standard 2-tailed *t* tests for continuous variables.

c Adjusted for all demographics, education level, and variables associated with high adherence at the *P* = .10 level in bivariable analyses. APRs, 95% CIs, and *P* values based on Poisson regression models with log link and robust variance.

d Including other state- or city-sponsored free or low-cost insurance.

e Defined as use of any social networking sites such as Facebook, MySpace, or Twitter.

f Summed score based on following yes/no items: controlling or losing weight, reducing stress, eating healthfully, reducing salt intake, exercising, reducing alcohol use, stopping smoking, taking medication, other dietary changes, visiting a medical professional.

g Assessed by using the Centers for Disease Control and Prevention’s Health-Related Quality of Life scale (14).

### Demographic, socioeconomic, and health care–related factors

No demographic variables were significantly associated with high adherence among younger adults in bivariable analyses. In older adults, age (mean age [standard deviation] in those with high adherence [69.7 y, (7.2 y)] vs low or medium adherence [68.7 y, (6.9 y)], *P* = .05) and Spanish (35.9%) versus English (28.1%, *P* = .08) language of interview were positively associated high adherence. Of the socioeconomic/health care–related factors, education level (>high school, 46.7%; high school diploma/GED, 41.0%; some high school [no diploma], 40.2%; vs ≤8th grade, 29.2%; *P* = .01), insurance status (private or Medicare, 57.1%; uninsured, 41.2%; vs any Medicaid, 31.4%; *P* < .001), and employment status (unemployed, 36.3% vs employed, 28.1%; *P* = .04) were positively associated with high adherence. Computer literacy was positively associated with high adherence in both younger (41.7% vs 22.8% not computer literate; *P* = .01) and older (57.6% vs 32.2% not computer literate; *P* = .003) adults.

In younger adults, none of the demographic or socioeconomic status or health care–related factors remained significant in multivariable analyses. In older adults, age (APR = 1.02; 95% CI, 1.00–1.04; *P* = .02), education (>high school diploma vs ≤8th grade, APR = 1.51; 95% CI, 1.06–2.14; *P* = .02) and insurance status (private or Medicare vs any Medicaid, APR = 1.67; 95% CI, 1.18–2.38; *P* = .004) remained positively related to high adherence. 

### Patient-related factors

In bivariable analyses, only in younger adults were typical alcohol consumption (1 drink/d, 23.1%; ≥2 drinks/d, 16.4%; vs nondrinkers, 27.5%; *P* = .08) and smoking status (current smoker, 46.7% vs never/former smoker, 22.7%; *P* = .003) related to high adherence. Among older adults, only blood pressure control knowledge score (high score, 46.3% vs low score, 24.4%; *P* < .001) and physical activity (active, 54.1% vs inactive, 32.6%; *P* = .007) were positively associated with high adherence. 

In multivariable analyses, alcohol consumption remained negatively associated with high adherence (≥2 drinks/d vs nondrinker, APR = 0.58; 95% CI, 0.35–0.95; *P* = .03) in younger adults. In older adults, only hypertension control knowledge positively predicted high adherence (high vs low, APR = 1.70; 95% CI, 1.30–2.22; *P* < .001).

### Condition-related factors

In bivariable analyses, in younger adults duration of hypertension was negatively associated with high adherence (5 to <10 y, 26.7%; ≥10 years, 19.9%; vs <5 y, 34.4%; *P* = .03). Among HRQOL measures, recent poor physical health was negatively associated with high adherence in younger (yes, 14.1% vs no, 31.9%; *P* < .001) and older adults (yes, 20.3% vs no, 42.7%; *P* < .001). Similarly, recent poor mental health was negatively related to adherence in younger adults (yes, 16.0% vs no, 26.6%; *P* = .06) and older adults (yes, 17.0% vs no, 38.5%, *P* < .001). Poor health–related activity limitation was negatively related to high adherence only in older adults (yes, 12.8% vs no, 38.5%; *P* < .001). Conversely, in younger adults, those with poorer self-rated general health had a higher prevalence of high adherence (fair/poor, 38.6%; good, 31.3%; vs very good/excellent, 17.4%; *P* < .001).

In multivariable analyses, duration of hypertension (≥10 y vs <5 y, APR = 0.54; 95% CI, 0.34–0.84; *P* = .006) remained negatively related to adherence in younger adults. Among HRQOL domains, recent poor physical health remained negatively associated with high adherence in both younger (APR = 0.53; 95% CI, 0.33–0.87; *P* = .01) and older (APR = 0.51; 95% CI, 0.35–0.74; *P* < .001) adults. Poor health–related activity limitation (APR = 0.35; 95% CI, 0.17–0.70; *P* < .001) remained negatively related to high adherence in older adults. In younger adults, those with poor or fair self-rated health versus very good or excellent self-rated health had a higher prevalence of high adherence (APR = 2.37; 95% CI, 1.54–3.63; *P* < .001).

## Discussion

We found low levels of high adherence to antihypertensive medication in a sample of urban community-dwelling Hispanics. Finding were consistent with those in previous research (2,7); adherence estimates from our sample (less than a quarter of younger adults and approximately one-third of older adults) were substantially lower than estimates from previous studies in non-Hispanic community-dwelling adults with hypertension (ranging from 36% to 58% in studies using the MMAS-8) (17–19). These disparities in adherence may in part explain the documented lower rates of hypertension control in US Hispanics (3). Healthy People 2020 sets national population-level hypertension and CVD goals, including targets for adherence to prescribed medications among adults with hypertension (20). Our results suggest that equitable achievement of these national goals will require particular attention to suboptimal antihypertensive medication adherence found in this study and other samples of US Hispanics, particularly in younger adults.

This study used the WHO Multidimensional Adherence Model to systematically assess predictors of antihypertensive medication adherence by age (4). Our finding were consistent with findings from previous studies, in which younger adults were significantly less likely to have high adherence than were older adults (5,7,18). Within age group–specific analyses, however, advancing age predicted adherence only among older adults. Conversely, a longer duration of hypertension, independent of age, predicted lower adherence only among younger adults, a finding consistent with longitudinal findings that antihypertensive therapy persistence (continued adherence with recommended therapy) declines over time to a greater degree in newly diagnosed younger adults than in older adults (21). This finding may indicate the role of declining motivation to persistently adhere to medication over time in younger people with hypertension, potentially given the asymptomatic nature of hypertension and the later age of manifestation of chronic CVD. Taken together with our findings, this finding suggests that duration of illness may be a stronger predictor of adherence in younger adults, whereas advancing age may be more predictive of adherence in older adults, perhaps coinciding with better familiarity and acceptance of aging-related processes.

Socioeconomic and health care–related variables were predictive of high adherence only in older adults. A higher education level predicted adherence only in older adults. Although previous research is inconsistent regarding age-related variation in the association between education and health, our results support the cumulative advantage hypothesis, which suggests that educational disparities in health are larger in older individuals (22,23). This may indicate that the cognitive, psychosocial, and material resources afforded by better education are deployed for better adherence more effectively by older than by younger adults (23). Our findings were also consistent with findings in prior research with older adults, in which having private insurance or Medicare only was associated with better adherence than having any Medicaid (24). Conversely, insurance status was not related to adherence in younger adults. Research has shown significantly higher levels of cost-related nonadherence to antihypertensive medications in younger adults, irrespective of coverage — possibly because of competing expenditure priorities during this life stage (25). This finding may mean that treatment cost burden, a factor we did not assess, independent of insurance status may be a more relevant barrier to adherence in this age group and especially in our low-income sample. Future efforts must consider not just access barriers but also higher out-of-pocket medication expenses, a main contributor to cost-related nonadherence in older and especially younger adults (25,26).

In terms of patient factors, our findings were consistent with findings of prior literature; increasing levels of alcohol consumption negatively predicted high adherence in younger adults but knowledge of blood pressure control positively predicted high adherence in older adults (27). Interventions targeting alcohol behaviors in younger adults must consider potential mechanisms, including alcohol’s role in promoting forgetfulness, willful nonadherence to avoid drug interactions, or maladaptive coping in young people facing serious chronic conditions (27). The positive relationship between blood pressure control knowledge and adherence in older adults alone may highlight the importance of superior knowledge and cognition in aging populations (4,6). However, education-based efforts at improving patient knowledge must be sustained for long periods while also combining them with other evidence-based strategies (8).

Finally, among condition-related factors, in younger adults, inconsistencies were observed in the relationship between domains of HRQOL and high adherence. Consistent with findings of some other studies, our finding was that those with better self-rated general health had a lower prevalence of adherence, suggesting that in younger adults, self-appraisal of general health as “excellent” or “very good” may trigger nonadherence, possibly given that many in this age group have not developed physical CVD symptoms yet (28,29). Conversely, recent poor physical health also predicted a lower prevalence of high adherence, suggesting that physical disease symptoms may be associated with reduced adherence. Therefore, different domains of HRQOL may have distinct influences on adherence in younger adults, and future research must explicitly understand the mechanisms by which specific aspects of HRQOL influence adherence in this age group (29,30). In older adults, both poor physical health and poor health–related activity limitation were associated with lower prevalence of adherence, indicating that aging-related physical health symptoms and associated disabilities may be important barriers to adherence in older populations. On the other hand, in both younger and older adults, recent poor mental HRQOL was significantly related to high adherence in bivariable but not in adjusted analyses. Although prior research has been largely inconsistent regarding the role of mental health HRQOL measures in predicting adherence (29,30), clinical measures of mental health (eg, depression, depressive symptoms) have consistently been documented as predictors of adherence (6), including among Hispanics (28). Future research must delineate the relevance of specific measures of mental health (HRQOL measures vs clinical measures) in relation to adherence.

This study addresses important gaps in the literature. Specifically, our systematic assessment identified several previously unevaluated factors associated with antihypertensive medication adherence in an understudied population using a large sample of Hispanics and validated measures (6,8). However, our study has limitations. The cross-sectional nature of this study precludes causal claims regarding the relationship between the identified predictors and adherence. Longitudinal studies of adherence among recently diagnosed individuals would be useful in confirming our results. Also, there is potential for response bias because of self-reported data. However, this risk is minimal, given that the survey used widely validated measures and standardized protocols for administration. The study population comprised mostly first-generation immigrants born in the Dominican Republic residing in northern Manhattan, limiting generalizations to other US Hispanic subgroups (3). Conducting similar studies in other Hispanic subgroups would be fruitful. Finally, the broad categorization of younger adults as those younger than 60 years may mask further age differences in predictors of adherence within this age group. However, sensitivity analyses found no differences in patterns of predictors across even younger ages (<50 y and 50–59 y) (Appendix, Supplementary Table 2).

Our finding of suboptimal antihypertensive medication adherence should be considered in conjunction with evidence of lower levels of hypertension awareness, diagnosis, and treatment among US Hispanics (3). This evidence suggests that addressing adherence, while critical, can form only part of the efforts to tackle ethnic disparities in hypertension control. Younger adults were less likely to have high adherence, and evidence of declining adherence with longer duration in this group indicates the need to target persistent adherence in these individuals to ensure hypertension control. Younger and older people with hypertension shared few predictors of adherence. Interventions to improve adherence targeting socioeconomic and health care resources, aging-related physical health and activity limitations, and educational efforts to improve knowledge may be better suited for older Hispanics with hypertension, whereas efforts targeting risky behaviors and specific domains of HRQOL may be more relevant among younger Hispanics with hypertension. At the very least, evidence of these age-related differences highlights the importance of developing approaches tailored to the specific life stage of people with hypertension.

## Appendix Supplementary Tables

This appendix is available for download as a Microsoft Word document.
