# Supplementary material for: Age-Related Differences in Antihypertensive Medication Adherence in Hispanics: A Cross-Sectional Community-Based Survey in New York City, 2011–2012
Source: Prev Chronic Dis. 2017 Jul 13;14:E57. doi: 10.5888/pcd14.160512 (PMC5510304; doi:10.5888/pcd14.160512)
Supplement: Supplementary file 1 [file 16_0512Appendix.docx]

Supplemental Table 1. Sample characteristics among Hispanic adults with hypertension by age group, WICER Survey, New York City, 2011-2012

|  | Younger Adults (<60 years) | Older adults (≥60 years) |  |
| --- | --- | --- | --- |
|  | % (N) or Mean (SD) | % (N) or Mean (SD) | p value ^b^ |
| ***Socioeconomic/Health Care factors*** |  |  |  |
| Insurance status |  |  |  |
| Any Medicaid, including other State or City sponsored free or low-cost insurance | 81.7 (348) | 88.1 (538) | <0.001 |
| Private or Medicare only | 9.6 (41) | 9.2 (56) |  |
| Uninsured | 8.7 (37) | 2.8 (17) |  |
| Current employment status |  |  |  |
| Employed | 59.7 (255) | 36.5 (223) | <0.001 |
| Unemployed | 40.3 (172) | 63.5 (388) |  |
| Household composition |  |  |  |
| Lives with ≤ 2 people or alone | 40.3 (172) | 63.5 (388) | <0.001 |
| Lives with >2 persons | 59.7 (255) | 36.5 (223) |  |
| Computer literacy |  |  |  |
| No | 91.5 (386) | 94.5 (571) | 0.054 |
| Yes | 8.5 (36) | 5.5 (33) |  |
| ***Patient factors*** |  |  |  |
| BP control knowledge score |  |  |  |
| Low (at or below median) | 57.6 (247) | 56 (344) | 0.619 |
| High (above median) | 42.4 (182) | 44 (270) |  |
| Weight status (BMI) |  |  |  |
| Normal weight (<25 kg/m2) | 12.7 (54) | 16.7 (102) | 0.189 |
| Overweight (25.0-29.9 kg/m2) | 38.4 (163) | 38 (232) |  |
| Obese (> 30.0 kg/m2) | 48.8 (207) | 45.3 (276) |  |
| Alcohol use on typical occasion |  |  |  |
| Non-drinker | 59.4 (247) | 78.8 (469) | <0.001 |
| 1 drink | 15.6 (65) | 10.6 (63) |  |
| >=2drinks | 25 (104) | 10.6 (63) |  |
| Smoking status |  |  |  |
| Never/Former smoker | 92.8 (387) | 96.5 (586) | 0.007 |
| Current smoker | 7.2 (30) | 3.5 (21) |  |
|  |  |  |  |
| Physical Activity |  |  |  |
| Active | 13.1 (54) | 6.3 (37) | <0.001 |
| Inactive | 86.9 (357) | 93.7 (553) |  |
|  |  |  |  |
|  |  |  |  |
|  |  |  |  |
| ***Cont’d*** |  |  |  |
| ***Condition factors*** |  |  |  |
| Duration with hypertension |  |  |  |
| <5 years | 22.7 (90) | 12.6 (66) | <0.001 |
| 5 to <10 years | 26.5 (105) | 14.2 (74) |  |
| >=10 years | 50.9 (202) | 73.2 (382) |  |
| Comorbid conditions |  |  |  |
| None | 63.7 (270) | 51.5 (308) | 0.001 |
| 1 | 28.1 (119) | 36.8 (220) |  |
| ≥ 2 | 8.3 (35) | 11.7 (70) |  |
| Physically unhealthy days |  |  |  |
| Low (at or below median) | 58 (235) | 61.4 (361) | 0.287 |
| High (above median) | 42 (170) | 38.6 (227) |  |
| Mentally unhealthy days |  |  |  |
| Low (at or below median) | 81.9 (339) | 80.3 (480) | 0.52 |
| High (above median) | 18.1 (75) | 19.7 (118) |  |
| Health-related activity limitation days | |  |  |
| Low (at or below median) | 85.3 (349) | 81.7 (486) | 0.129 |
| High (above median) | 14.7 (60) | 18.3 (109) |  |
| Self-rated health |  |  |  |
| Excellent/Very good | 57.5 (242) | 48.7 (288) | 0.022 |
| Good | 22.8 (96) | 28.1 (166) |  |
| Fair/Poor | 19.7 (83) | 23.2 (137) |  |

Abbreviations – WICER, Washington Heights/Inwood Informatics Infrastructure for Community-Centered Comparative Effectiveness Research; SD, Standard Deviation; GED, General Educational Development test; NVS, Newest Vital Sign Score; BP, Blood Pressure; BMI, Body Mass Index;

^a^ High adherence (values of 8) and low or medium (0 to <8) on 8-item Morisky Medication Adherence Scale (MMAS-8)

^b^ p-value based on chi-square test for categorical variables and standard 2-tailed t-tests for continuous variables

Supplemental Table 2. Bivariable predictors of antihypertensive medication adherence among those <50 years vs. 50-59 years, Hispanic adults with hypertension, WICER Survey, New York City, 2011-2012

|  | **>50 years** | |  | **50-59 years** | |
| --- | --- | --- | --- | --- | --- |
|  | **(%) or Mean (SD)** | **p value^c^** |  | **(%) or Mean (SD)** | **p value^c^** |
| ***Demographics*** |  |  |  |  |  |
| Age (years) | 42.8 (5.8) vs. 43.6 (6.0) | 0.51 |  | 55.2 (2.8) vs. 55.6 (2.6) | 0.31 |
| Sex |  |  |  |  |  |
| Male | 20.0 | 0.36 |  | 23.3 | 0.84 |
| Female | 27.6 |  |  | 24.5 |  |
| Immigration status |  |  |  |  |  |
| Living in U.S. for <10 years | 33.3 | 0.42 |  | 22.2 | 0.77 |
| Living in the U.S. ≥10 years or US. born | 24.3 |  |  | 24.4 |  |
| Language of interview |  |  |  |  |  |
| English | 18.4 | 0.25 |  | 21.9 | 0.52 |
| Spanish | 28.1 |  |  | 25.2 |  |
| Marital Status |  |  |  |  |  |
| Divorced/Separated/Unmarried | 28.4 | 0.26 |  | 24.3 | 0.10 |
| Married or cohabiting | 19.6 |  |  | 24.4 |  |
| ***Socioeconomic/Health Care factors*** |  |  |  |  |  |
| Educational level |  |  |  |  |  |
| ≤8 grade | 17.1 | 0.21 |  | 27.0 | 0.61 |
| 9-11 years | 19.4 |  |  | 18.0 |  |
| High school/GED | 39.1 |  |  | 24.6 |  |
| > High school | 29.7 |  |  | 25.8 |  |
| Insurance status |  |  |  |  |  |
| Any Medicaid, including other State or City sponsored free or low-cost insurance | 21.1 | 0.15 |  | 24.1 | 0.52 |
| Private or Medicare only | 26.7 |  |  | 19.2 |  |
| Uninsured | 43.8 |  |  | 33.3 |  |
| Current employment status |  |  |  |  |  |
| Employed | 26.7 | 0.48 |  | 21.1 | 0.28 |
| Unemployed | 20.6 |  |  | 27.0 |  |
| Household composition |  |  |  |  |  |
| Lives with ≤ 2 people or alone | 29.0 | 0.46 |  | 26.9 | 0.28 |
| Lives with >2 persons | 22.7 |  |  | 21.6 |  |
| Computer literacy |  |  |  |  |  |
| No | 23.2 | 0.22 |  | 22.7 | 0.02 |
| Yes | 37.5 |  |  | 45.0 |  |
| ***Patient factors*** |  |  |  |  |  |
| BP control knowledge score |  |  |  |  |  |
| Low | 20.3 | 0.13 |  | 24.9 | 0.75 |
| High | 32.1 |  |  | 23.3 |  |
| Weight status (BMI) |  |  |  |  |  |
| Normal weight  (<25 kg/m2) | 23.5 | 0.90 |  | 16.2 | 0.38 |
| Overweight  (25.0-29.9 kg/m2) | 27.7 |  |  | 23.3 |  |
| Obese (>30.0 kg/m2) | 24.2 |  |  | 26.9 |  |
| Alcohol consumption on typical occasion |  |  |  |  |  |
| Non-drinker | 30.3 | 0.10 |  | 26.5 | 0.40 |
| 1 drink/day | 27.3 |  |  | 20.9 |  |
| ≥2drinks/day | 11.4 |  |  | 18.8 |  |
| Smoking status |  |  |  |  |  |
| Never/Former smoker | 23.9 | 0.60 |  | 22.2 | 0.002 |
| Current smoker | 33.3 |  |  | 50.0 |  |
| Physical Activity |  |  |  |  |  |
| Inactive | 27.0 | 0.67 |  | 22.2 | 0.07 |
| Active | 22.2 |  |  | 36.1 |  |
| ***Condition factors*** |  |  |  |  |  |
| Duration with hypertension |  |  |  |  |  |
| <5 years | 25.6 | 0.26 |  | 42.6 | <0.001 |
| 5 to <10 years | 15.2 |  |  | 31.9 |  |
| ≥10 years | 31.7 |  |  | 16.8 |  |
| Comorbid conditions |  |  |  |  |  |
| None | 28.6 | 0.20 |  | 26.7 | 0.36 |
| 1 | 11.5 |  |  | 19.4 |  |
| ≥ 2 | 33.3 |  |  | 28.1 |  |
| Poor physical health, 30-day |  |  |  |  |  |
| No | 30.3 | 0.07 |  | 32.7 | 0 |
| Yes | 15.6 |  |  | 13.6 |  |
|  |  |  |  |  |  |
| Poor mental health, 30-day |  |  |  |  |  |
| No | 26.6 | 0.22 |  | 26.5 | 0.128 |
| Yes | 12.5 |  |  | 17.0 |  |
| Poor health-related activity limitation, 30-day |  |  |  |  |  |
| No | 26.8 | 0.37 |  | 25.3 | 0.557 |
| Yes | 15.4 |  |  | 21.3 |  |
| Self-rated health |  |  |  |  |  |
| Very good/Excellent | 18.4 | 0.08 |  | 16.9 | 0.002 |
| Good | 30.8 |  |  | 31.4 |  |
| Fair/Poor | 40.9 |  |  | 37.7 |  |
